# Passes allowed per defensive action: a measure of pressing or defensive activity?

**DOI:** 10.3389/fspor.2026.1972815

**Published:** 2026-10-30

**Authors:** Bochan Kim, Nic James, Goran Sporiš, Ben Carter, Aled James, Seamus Brady, Jongwon Kim, Nimai Parmar, Ungho Gwon, Ahmet Naci Dilek, Goran Vučković

**Affiliations:** 1 London Sport Institute, School of Science & Technology, Middlesex University, London, United Kingdom; 2 Faculty of Kinesiology, University of Zagreb, Zagreb, Croatia; 3 Department of Health, Exercise Science and Recreation Management, University of Mississippi, Oxford, MS, United States; 4 School of Engineering and Applied Sciences, Swansea University, Swansea, United Kingdom; 5 Faculty of Sport Science, Bartın University, Bartın, Türkiye; 6 Faculty of Sport, University of Ljubljana, Ljubljana, Slovenia

**Keywords:** defensive strategy, football, passes allowed per defensive action, performance analysis, PPDA

## Abstract

The high press is a collective out of possession strategy designed to reduce the time and space available to opponents in possession, in the opponent's half of the pitch. To measure the high press, player and ball movements need to be tracked, and a complex algorithm developed. Consequently, a proxy measure for the high press was developed, passes allowed per defensive action (PPDA) which could be calculated without recourse to x y coordinate data, a resource only available for the highest-level football competitions. PPDA values are under reported in scientific papers and their utility unknown. The objective was to consider PPDA in relation to defensive activity encapsulated within a playing style framework. All matches in the 2017/18 English Premier League (EPL) were accessed through Opta Sportsdata. Variables analysed using Spearman's Rho, Wilcoxon signed-ranks and Factor Analysis were PPDA, ball recoveries in the middle and attacking thirds, progressive passes and possession percentage for home and away teams classified as highest, middle and lowest ranked teams. Teams with the lowest median PPDA tended to finish higher in the league (PPDA related to points gained at home (Spearman's Rho = −0.52) and away (−0.57), with colloquially recognised high press teams demonstrating low average PPDA values. PPDA was associated with home advantage, but relative team strength had a greater effect with stronger teams having lower PPDA values than weaker teams, irrespective of venue (medium and large effect sizes for Wilcoxon signed ranks tests except when the lowest ranked teams played at home against the middle-ranked teams (*p* = 0.8). High possession teams tended to play a low percentage of progressive passes and a low PPDA, indicative of an indirect style of play, against an opponent with a high PPDA, suggesting a low block defence. Individual teams displayed large variations in PPDA values suggesting PPDA, in conjunction with other metrics, could be informative for describing defensive playing style, which is dependent on match context, evident here in matches where possession was unequally shared. Future research should consider the utility of PPDA in closely contested matches but in conjunction with spatial-temporal tracking metrics.

## Introduction

Football is characterised by complex tactical demands, as players adapt to differing roles and responsibilities due to the continuously changing game situation (2). This includes repeated transitions between possession, non-possession and set-piece phases, requiring teams to adapt their tactical behaviours according to the changing location of the ball, the opponent's structure and positioning, and the match context (3). However, a large proportion of performance analysis related football research has focused on attacking play and in particular, on-ball indicators such as possession, passing, shooting and attacking effectiveness (4, 5). Whilst these actions describe what the team in possession does, they provide a limited account of the team without possession i.e., off-ball movements and actions. This can be viewed as problematic due to football being a low scoring sport (6, 7) and it is thus logical to infer that defensive strategies and off-ball movements play an important role in the dynamics of the game (8, 9) with the ability to organise the defence, regain possession and provide pressure to opponents all influencing match outcomes.

A multifaceted approach to analysing football performance is clearly logical although directly measuring off the ball behaviours to assess defensive strategies such as pressing intensity i.e., frequency and aggression of defensive engagement, will require accurate player and ball locations, interpersonal distances, collective movements of the defending team etc. Tracking (player movement) data will therefore enable more accurate and insightful analyses; however, this requires specialist video, data, processing time and technical expertise. Recent studies adopting this approach have found that success in elite international football may be determined by a multifactorial interaction of offensive efficiency, territorial progression, spatial duel distributions and key situational factors such as scoring first and goal timing (10). Similarly, defensive pressure measures differed according to tournament and match outcome measures, when comparing FIFA World Cup 2022 and FIFA Club World Cup 2025 data, suggesting both multifactorial and context-specific affects (11). Praça et al. (7) integrated positional data with match events to evaluate the tactics of teams playing in the FIFA World Cup 2022 and found that multiple defensive strategies could lead to winning performances although an aggressive style aimed at forcing turnovers high up the pitch was the most successful.

Access to tracking data is usually restricted to well-resourced and funded teams and leagues, thus simpler event-data metrics may provide useful insights into pressing i.e., spatial defensive strategy involving advanced defensive pressure, but there are limitations. Passes allowed per defensive action (PPDA) was introduced by Trainor (1) as a proxy for the intensity of the high press i.e., pressing high up the pitch. Anecdotally, football pundits often use PPDA values as a direct measure of the high press, however this belief has not been scientifically validated. It is calculated by dividing the number of passes completed by the team in possession in a defined zone, by the number of defensive actions made by the out of possession team in that same zone. A lower PPDA value indicates that the defending team allowed fewer opponent passes before making an on the ball defensive action. In applied settings, it is convention that values below approximately 8–10 are interpreted as consistent with more aggressive pressing, with very low values suggesting frequent advanced defensive engagement. For example, Manchester City's median PPDA in the present EPL dataset was approximately 8, which aligns with their widely recognised high-possession, high-pressing style. Whilst this is illustrative rather than validation of PPDA, low PPDA values have been generally interpreted as evidence that a team is high pressing, whereas high PPDA values being interpreted as the opponents having more possession with less pressure. Given that these descriptions align with defensive playing styles, for example, when the defending team have retreated into a low block i.e., pressure only applied to opponents when the ball is close to, or inside the penalty box, PPDA may be more indicative of playing style than the more precise expression of the high press.

The categorisation of five key phases of play (3) has been delineated e.g., an established defence may consist of different defensive strategies, such as a high press which refers to a collective team action typically involving midfield and attacking players closing down the space (exerting pressure) available for the opposition in an effort to restrict passing options (12) and also to regain possession in an advanced area of the pitch (7, 13). An additional consideration that determines a successful defensive strategy is the timing of a press (14). Exerting pressure quickly after losing possession (i.e., defensive transition) is often referred to as counter pressing, which consists of an immediate attempt to win the ball back (Pep Guardiola's Barcelona had to regain possession within 6 s) usually high up the pitch, to prevent the opponent's quick attacking intention (14, 15). The aim is not simply to tackle the ball carrier, but to reduce the time available for the ball carrier to make a pass and close down the space available to the ball carrier and potential ball receivers (16). This potentially forces hurried decisions and creates turnovers or recoveries in areas from which attacks/counter attacks can be launched quickly. Set pieces are tactically distinct, because the defensive and attacking structures are generally pre-organised and do not always reflect the same open-play pressing decisions. Consequently, any whole-match metric that aggregates actions across open play, transitions and set pieces risks mixing different “moments of the game” into a single value. This is especially relevant when interpreting PPDA, because a low value may reflect sustained high pressing, frequent counter-pressing after possession losses, match dominance that pins the opponent back, or a combination of these behaviours. These observations suggest that PPDA needs to be considered within a wider contextually specific framework to better understand how the PPDA metric was achieved and what it thus represents.

Despite its rising use in applied and academic contexts, PPDA has not been fully validated as a direct measure of high pressing. It is an event-based ratio rather than a behavioural measure of pressure. It does not record players who close space down without making a defensive action, nor does it identify whether a team intended to press. Therefore, unsuccessful pressing and no attempt to press can appear similar. Equally, a high PPDA value may indicate a deliberate low block, passive defending, tactical protection of defensive spaces, or simply an opponent's ability to hold on to possession (challenged or unchallenged).

The spatial definition of PPDA also requires some scrutiny. Traditionally PPDA is calculated in a zone corresponding to the attacking 60% of the pitch from the defending team's perspective, rather than only the final third (Figure 1). This is important due to coaching language which often distinguishes high, mid and low blocks according to “thirds” of the pitch. A defensive action in the 40%, 50% or 60% region of the pitch may therefore represent different tactical behaviours. As a result, PPDA may be more appropriately understood as an indirect measure of defensive activity or pressing intent, rather than as a direct measure of high pressing, particularly since “high” is widely interpreted as the final third. Turnovers, ball recoveries and PPDA may be complementary rather than interchangeable metrics. Turnovers and recoveries identify where possession is regained, whereas PPDA describes the frequency of defensive actions relative to the opponent's passing volume in a defined zone. Used together, these indicators may help analysts describe whether a team actively seeks to regain possession high up the pitch, whether it allows the opponent to play before engaging, and whether its out-of-possession strategy is linked to a broader playing style. Considering football within a multidimensional framework that acknowledges attacking efficiency and defensive organisation are intrinsically linked and contextually specific will facilitate the understanding of the value and importance of PPDA.

**Figure 1:**
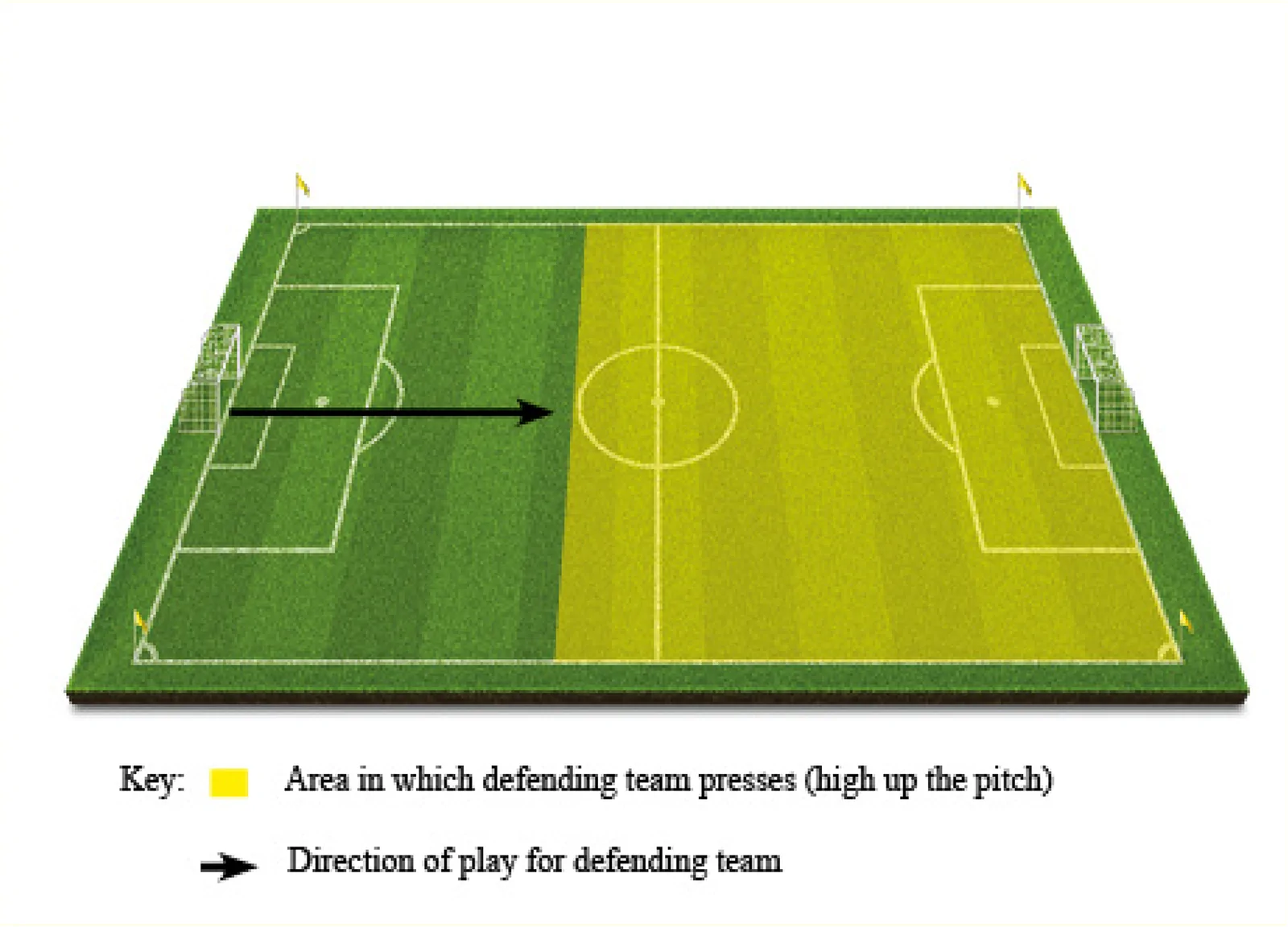
Area where actions take place to calculate Trainor's (2014) PPDA metric.

This study evaluates the utility of PPDA in the 2017/18 English Premier League. Specifically, whether PPDA values (for team and opposition) are associated with relative team strength and defensive playing style. The vagueness of the PPDA metric, it does not represent closing down space behaviour that does not involve a defensive action, collective movements and the timing of a defensive press are not measured, suggests that PPDA needs to be interpreted as a construct that relates to defensive activity, most likely in relation to the playing style of the team. Hence, possession and progressive passing metrics will be used alongside PPDA to determine playing styles and the utility of PPDA in this context.

## Methods

All matches played in the 2017/18 EPL (*n* = 380) were analysed. Teams were classified as highest (top 5 EPL as all qualified for European competitions), middle (6–17 EPL) and lowest ranked (18–20 EPL as relegated to the Championship). These cut-offs represent success and failure but can result in similar performing teams being allocated to different groups (teams near the cut-offs). However, these groups represent an ordinal classification albeit with unequal group sizes. Post-season performance classifications have been criticised on the basis that individual team performances can fluctuate throughout the season, but this method classifies all one team's performances into one group (17). This method, whilst imperfect, was deemed to be satisfactory for discerning general trends, all teams played each other home and away, whilst acknowledging that individual team analyses would be more meaningful at the applied level.

Data was provided by Opta Sportsdata and permission to publish the findings given. The calculation of PPDA, as devised by Trainor (1), and now presented by Hudl (https://www.hudl.com) was used in this study. Hence the number of completed passes by the team in possession in their defensive 60% of the pitch (Figure 1) was divided by the number of defensive actions [tackles (successful and unsuccessful), interceptions and fouls] made by the out of possession team in the same area of the pitch. All match events were aggregated into one PPDA calculation. Ethical approval for the study was provided by the sports science sub-committee of Middlesex University's ethics committee in accordance with the 1964 Helsinki declaration.

### Exploratory assessment of professional perceptions (face validity) of PPDA

Sixteen participants (13 Performance analysts, 3 coaches) with an average of 9.4 years' experience (SD = 3.56) working in professional football answered an online questionnaire. Participants were recruited via invitation emails to fill out a SurveyMonkey questionnaire. All questions were developed for the purpose of this study ensuring multiple choice answers to capture all potential responses. Open dialogue boxes were available to supplement answers with more in-depth insights. Coaching experience was evidenced by UEFA Pro (*n* = 3), A (*n* = 1), B (*n* = 8) and C (*n* = 4) licences. All participants used Wyscout, Statsbomb or both and described PPDA correctly although not all mentioned the pitch area involved. Half of the participants thought PPDA was an accurate measure but criticised it because it was unable to distinguish when a team decided to press as opposed to when they did not. Some respondents therefore preferred to use x y coordinate data from Statsbomb. Low PPDA values (below 10) were thought to indicate high pressing (*n* = 11) with high values suggesting that a team drops deeper to defend.

### Measurement issues for calculating PPDA

PPDA values for three Champions League matches were calculated for each 15-minute period by one of the authors. These time periods were selected as they are calculated by Opta but they are likely to be more sensitive to error due to the smaller time frame used for the PPDA calculation. The mean difference between these values and those provided by Opta was 0.2. Observational differences were attributed to the accuracy of resetting a new time period and to relevant actions being missed when replays were shown during television coverage. Differences in PPDA values were small when passes were missed but more dramatic when defensive actions were missed. Very small definitional errors were present due to some subjectivity around defensive actions, for example due.

ls won and lost. These observations suggest that PPDA can be collected sufficiently accurately for these analysis purposes.

### Statistics

Data was downloaded into Excel spreadsheets (Microsoft Office Plus 2019, Redmond, USA) and combined into one sheet. Situational variables were added for each PPDA value for team quality (ranked by league finishing position into 3 groups), opposition strength (opponent's rank) and match venue (home or away). Data was transferred to IBM SPSS Statistics (version 28) to undertake statistical tests and checks for errors in the data. Spearman's Rho (*p*) correlation coefficients were used to test linear relationships for individual (match level) and averaged PPDA (team level) values due to the presence of outliers. Outliers were retained for all analyses, being representative of the true nature of variance within the dataset. Wilcoxon signed-ranks tests (*z*) were used to compare home vs. away PPDA performance (match level), according to team and opponent quality. Effect sizes were calculated by dividing the *z* statistic by the square root of the sample size (18) where values above 0.2 were considered small effects, 0.5 medium and 0.8 large (19). Exploratory factor analysis with Varimax rotation was used to assess the utility of PPDA in a model to assess playing styles (match level).

## Results

### The variation in PPDA

PPDA values for home teams typically ranged between 9.79 (25th percentile) and 15.24 (75th percentile) with values over 23.42 deemed outliers [3rd quartile + (or 1st quartile −)1.5× the interquartile range]. Similarly, away teams' PPDA values typically ranged between 10.51 (25th percentile) and 17.46 (75th percentile) with values over 27.90 deemed outliers. There was a low correlation for home and away PPDA values (Spearman Rho = −0.25; ES = small; Figure 2) although larger PPDA values (>20) typically associated with lower PPDA values (<20) for the opposition.

**Figure 2:**
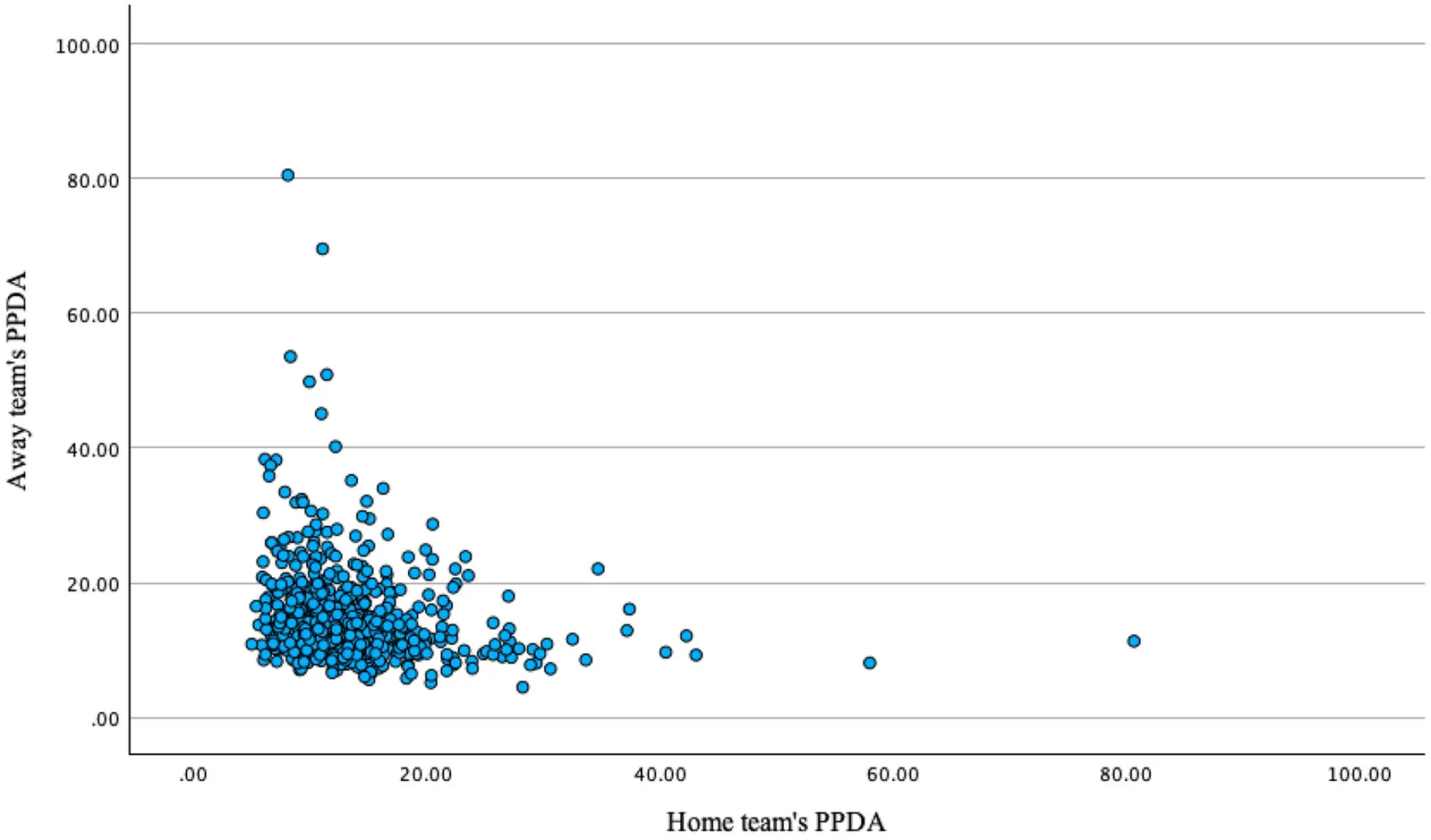
Scatterplot of PPDA values for home vs. away EPL teams.

The relationship between an EPL team's median PPDA over the full season and their opponents’ PPDA (Spearman's Rho = −0.62, ES = large) suggested that, of the top 5 ranked teams, only Chelsea and Manchester United, had average PPDA values that were similar to their opponents, which was similar to the rest of the teams in the league (Figure 3). The other highest ranked teams had lower median PPDAs than their opponents with Arsenal, who finished 6th, and were thus ranked in the middle category, the only other team with a lower PPDA value (median = 11.02) than their opponents (median = 16.82).

**Figure 3:**
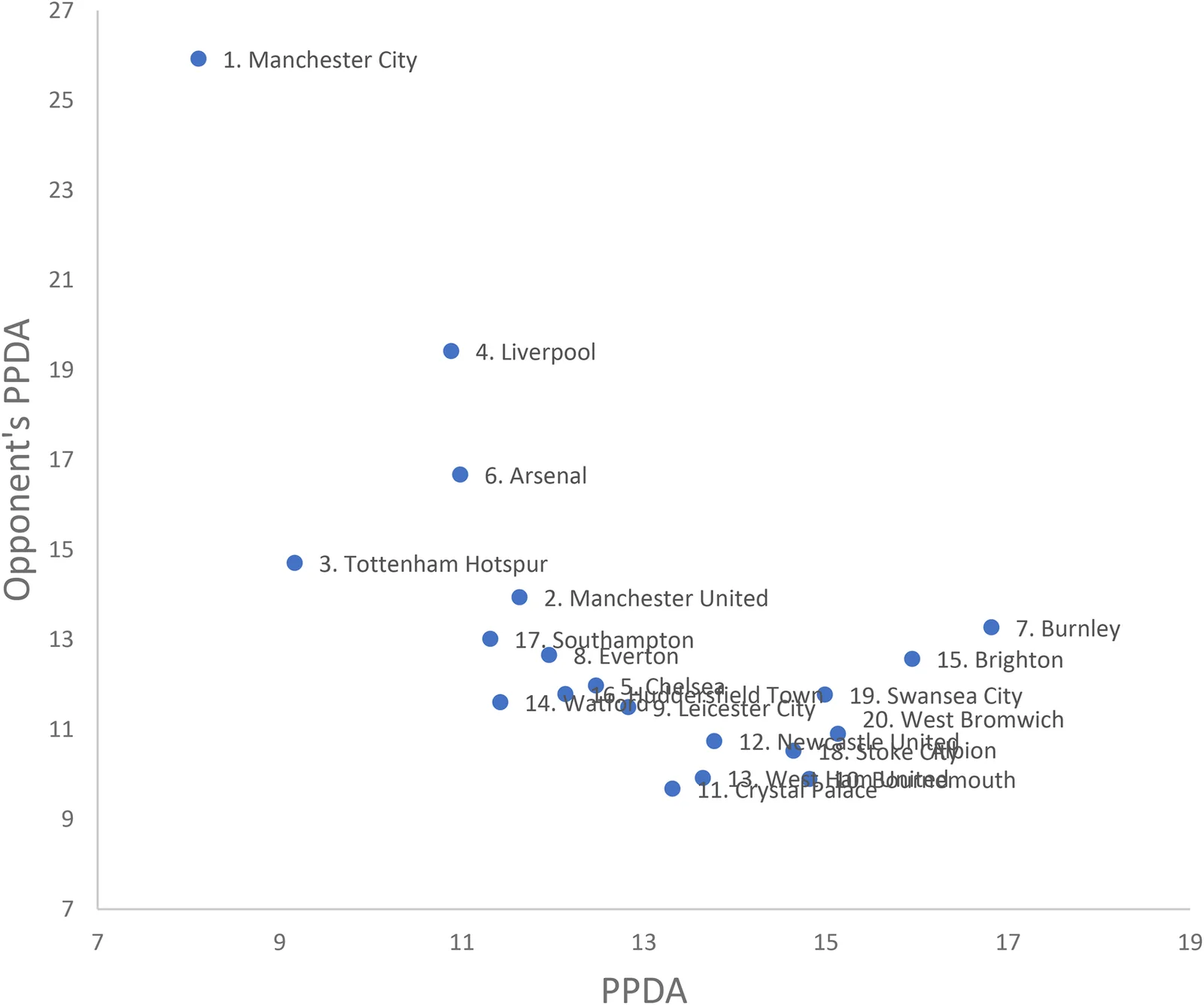
Scatterplot of median PPDA values over a season for EPL teams in relation to their opponents. Numbers represent finishing league position.

Teams with the lowest median PPDA over the whole season, tended to achieve more points (Spearman's Rho = −0.52, ES = large, for home PPDA; Spearman's Rho = −0.57, ES = large, for away PPDA). PPDA median values for individual teams were correlated (EPL Spearman's Rho = 0.88, ES = large) and tended to be slightly lower when playing at home compared to away in the EPL (*z* = −3.47, *p* < 0.001, ES = 0.56, large; Table 1).

**Table 1:** Median PPDA values for EPL teams playing home and away fixtures.

Position	EPL	Pts	PPDA	Away
			Home	
1	Manchester City^1^	100	8.08	8.44
2	Manchester United^1^	81	11.48	11.71
3	Tottenham^1^	77	8.96	9.61
4	Liverpool^1^	75	10.41	11.16
5	Chelsea^1^	70	11.45	13.17
6	Arsenal^2^	63	10.50	12.14
7	Burnley^2^	54	16.05	18.23
8	Everton^2^	49	10.68	12.97
9	Leicester^2^	47	11.35	13.14
10	Newcastle^2^	44	12.17	15.79
11	Crystal Palace^2^	44	11.43	14.03
12	Bournemouth^2^	44	14.85	14.79
13	West Ham^2^	42	12.59	14.95
14	Watford^2^	41	11.40	11.45
15	Brighton^2^	40	15.09	16.18
16	Huddersfield^2^	37	11.32	12.50
17	Southampton^2^	36	10.64	11.81
18	Swansea^3^	33	13.69	17.57
19	Stoke^3^	33	13.89	16.12
20	West Bromwich^3^	31	16.00	15.00
**Median**			**11**.**62**	**13**.**20**

1,2,3 Team ranking category.

PPDA values were influenced by both match venue and team rank with higher ranked teams having lower PPDA values than their lower ranked opponents, irrespective of playing home or away (*p* < .001, Table 2) except when the lowest ranked teams played at home against the middle-ranked teams (*p* = 0.8). Similarly ranks teams tended to have similar PPDA values when playing against each other although middle-ranked teams exhibited a small home advantage (*p* < .05).

**Table 2:** Home vs. away PPDA values for different team ranks.

Rank	Home team			Away team			Rank	Wilcoxon signed ranks	Sig.
	25th	**50th**	75th	25th	**50th**	75th			
Highest	9.84	**12**.**29**	14.99	8.46	**10**.**82**	15.41	Highest	0.24	0.81
	8.17	**9**.**87**	11.47	13.77	**19**.**45**	25.76	Middle	6.04	<.001^M^
	6.59	**9**.**08**	11.39	18.65	**23**.**11**	30.38	Lowest	3.24	<.001^L^
Middle	11.82	**15**.**96**	21.76	9.17	**10**.**85**	13.68	Highest	4.50	<.001^M^
	9.89	**11**.**34**	14.53	10.26	**12**.**91**	15.72	Middle	2.36	<.05^S^
	8.45	**10**.**40**	12.42	11.14	**14**.**00**	18.34	Lowest	3.90	<.001^M^
Lowest	17.18	**19**.**78**	22.24	8.04	**9**.**50**	12.95	Highest	2.61	<.01^M^
	11.09	**12**.**15**	16.52	10.84	**12**.**54**	15.64	Middle	0.25	0.80
	9.89	**11**.**84**	14.31	10.68	**11**.**43**	20.97	Lowest	0.52	0.60^S^

Effect size = ^S^small, ^M^medium, ^L^large.

### The role of PPDA in playing style determination

The Kaiser–Meyer–Olkin measure (KMO = 0.69) verified the sampling adequacy. The communalities after extraction were over 0.7 for five of the seven performance metrics. Bartlett's test of sphericity (*χ*^2^ = 953.47, df = 21, *p* < .001) indicated that the correlations between performance metrics were sufficiently large. Three components satisfied Kaiser's criterion, eigenvalues >1.0 and in combination explained 75.74% of the total variance (Table 3). The scree plot supported the retention of three factors with the rotated component matrix for the factor loadings identifying the performance metrics associated with each factor (Table 4).

**Table 3:** Eigenvalues for components and total variance explained.

Component	Initial eigenvalues			Extraction sums of squares loadings			Rotation sums of squares loadings		
	Total	% of variance	Cumulative %	Total	% of variance	Cumulative %	Total	% of variance	Cumulative %
1	3.082	44.024	44.024	3.082	44.024	44.024	2.118	30.264	30.264
2	1.141	16.303	60.326	1.141	16.303	60.326	2.098	29.975	60.239
3	1.079	15.409	75.736	1.079	15.409	75.736	1.085	15.497	75.736
4	0.593	8.470	84.206						
5	0.485	6.930	91.136						
6	0.464	6.633	97.769						
7	0.156	2.231	100						

**Table 4:** Rotated component matrix for the performance metrics.

Performance metrics	Component		
	1	2	3
Away PPDA	**0** **.** **841**	0.098	0.086
Home PPDA	−0.170	**−0**.**786**	−0.117
Home recoveries in final 1/3%	0.461	0.499	−0.433
Home recoveries in middle 1/3%	0.124	0.132	**0**.**931**
Home possession %	**0**.**639**	**0**.**673**	0.097
Home progressive passes %	**−0**.**864**	−0.090	−0.010
Away progressive passes %	−0.003	**0**.**862**	0.002

Factor loading in bold highlight a strong positive correlation.

Descriptions of factors were based on the combination of associated performance metrics. Factor 1 (indirect possession against an opponent making few attempts to regain possession high up the pitch) defines how much the home team dominates possession (high possession %) by playing sideways and backwards passes (low percentage of progressive passes), with their opponent seldom challenging for the ball high up the pitch (high PPDA for away team). A team with a high score on this factor would likely play a possession based, indirect style of play (low percentage of progressive passes) with the opponent in a low block, allowing sideways and backward passes, to try to maximise their ability to prevent penetrative runs and passes into the area near, or in, their own penalty area. A low score on this factor would indicate the team plays a more direct style of play (high % of progressive passes) having less possession than their opponent who is making lots of challenges high up the pitch (low PPDA). Factor 2 reflects the opponent's interacting style of play in response, or as an effect on, the other team's style of play, as indicated in Factor 1. Hence both factors 1 and 2 load on the home team's proportion of possession but relates to how much possession the team in question has i.e., if the home team has a high percentage of possession the away team has a low percentage. Consequently Factor 2 highlights that when the home team has a high percentage of possession, they also have a low PPDA suggesting their opponents plays a more direct style of football (high proportion of progressive passes, counter attacking). Factor 3 (midfield battle) determines how well a team dominates this crucial area of the pitch, sometimes referred to as positional play. A team with a high score on this factor tends to dominate possession in the middle of the pitch, which can facilitate creating attacking opportunities.

## Discussion

Performance analysis is increasingly moving from isolated technical indicators toward multidimensional models combining technical-tactical, physical, spatial and contextual variables. Current approaches increasingly integrate event data (passes, recoveries, turnovers, defensive actions), tracking data (player positioning, distances, velocities, defensive organisation) in reference to contextual variables (match status, opponent quality, venue). Furthermore, machine-learning approaches for tactical pattern recognition are becoming increasingly visible in academic literature. However, the accessibility, and applicability across competitions, of technical indicators supports the use of metrics such as PPDA.

From a practical perspective PPDA has been a consistent metric provided to football professionals and consumers for over 10 years. Within the coaching community our small sample of professionals suggest an acceptance of the utility of the metric, albeit without too much scrutiny in terms of its absolute accuracy. A more rigorous examination of the metric was therefore necessary.

PPDA median values (12.47) in the EPL (2017/18) were similar to those found by Trainor (1) where EPL averages ranged between 11.0 (2010/11) and 12.92 (2013/14). However, if Trainor's values were means, as opposed to medians, this would suggest that the PPDA values in this study (mean = 14.72) were higher than previously reported, potentially due to a different set of teams. The correlation between home and away team's PPDA values were small suggesting home advantage was not present but that a match-level relationship existed between the two team's PPDA values. Some matches did present large differences between teams indicating that when one team had high PPDA values, potentially playing in a low block, but this conjecture would need supporting evidence from other metric values, their opponents had a consequent low PPDA value, usually coinciding with a high percentage of the possession, again logical when one team defends deep. The high possession team would be able to commit most of their players high up the pitch as they try to pass the ball through the defensive lines. If they lost the ball in this situation they could easily attempt to quickly regain possession, often called a counter press, and would thus have a low PPDA value. It was not surprising therefore, that Pep Guardiola's Manchester City (Mdn = 8.11) and Mauricio Pochettino's Tottenham Hotspur (Mdn = 9.17), both renowned for high possession, high press styles of play, had markedly lower average PPDA values compared to most teams in the league. Manchester City's opponents did not appear to attempt many defensive actions high up the pitch (Opponent PPDA Mdn = 25.93) anything like against other EPL opponents, with Liverpool (Mdn = 19.42), Arsenal (Mdn = 16.67) and Tottenham Hotspur (Mdn = 14.71) the only other teams where opponent PPDA values averaged over 14.

As an indicator of success, teams with the lowest median PPDA values tended to achieve more points although this should not be interpreted causally. Two top ranked teams, Manchester United and Chelsea, had average PPDA values, being managed by José Mourinho and Antonio Conte respectively, both renowned for adopting defensively minded counter attack playing styles. Similarly, two middle ranked teams, Southampton (Mauricio Pellegrino) and Watford (Marco Silva/Javi Gracia), were known for their possession-based football, had relatively low PPDA values, which went against the general trend. These examples show that PPDA can capture one dimension of playing style but does not, by itself, define tactical effectiveness. On this basis, PPDA cannot be seen as an indicator of success, at least for this elite level of football, since individual teams may utilise different strategies, such as using a low possession (low block) fast transition style of play and still be successful, the most notable example being the 2015/16 EPL champions Leicester City. A pragmatic view should emphasise that football performance always relates to team quality, possession dominance and territorial control, but opponent characteristics may simultaneously influence both PPDA and competitive performance.

Further examination of the much-cited home advantage demonstrated that teams tended to have lower PPDA values at home than away (large effect size) although the difference was small (Mdn difference = 1.58). However, when team and opponent rank were considered, higher ranked teams often recorded lower PPDA values than lower ranked opponents, irrespective of match venue. This was particularly true for the highest ranked teams suggesting that team quality and the ability to dictate territory and possession is more influential than match venue alone. The exploratory factor analysis supported this idea as it clearly identified statistical patterns indicating the interaction of playing styles between two competing teams. The basic pattern when one team had superior possession consisted of the high possession team playing indirect football (low proportion of progressive passes) with a low PPDA (high number of defensive actions, likely when their opponent regained possession high up the pitch). The opponent in this situation is likely to be in a low block formation, their high PPDA values support this conjecture, but validated tracking data measures are needed to support this suggestion. A low possession team, when gaining possession in their defensive areas, against a team with most of their players high up the pitch, will tend to play direct, a high proportion of progressive passes. The factor analysis supported this view and tends to suggest that the utility of PPDA is as a descriptive measure, within a contextual framework, of playing style.

The main limitation of PPDA is that it is a broad measure encompassing different events that would be better measured separately. It does not reflect players closing down space without making a positive defensive action, failed pressing attempts, the timing of pressure events, collective team movements, or whether any actions occurred during established defence, transition or set piece situations. However, very high PPDA values strongly suggest a low block defensive strategy resulting in opponent possession dominance. In comparison, very low PPDA values suggest possession dominance with the ball usually located high up the pitch against an opponent in a low block. These observations need to be verified using tracking data and the utility of such analyses made more practical if the different moments of the game (3) are separated for analyses. Playing styles are likely to correlate highly with match status, e.g., where a winning team becomes more defensively oriented (low block), particularly towards the end of a match, allowing the losing team more possession. Future research should validate PPDA against tracking-data pressure measures, analyst-coded pressing events and phase-specific analyses to determine whether low PPDA values reflect effective pressing, distinct from counter pressing, and the extent to which possession dominance affects the PPDA metric.

Contemporary football analysis increasingly recognises match performance as the interaction between attacking efficiency, defensive organisation, pressing intensity, transition behaviour and spatial control. Consequently, defensive metrics such as PPDA should be interpreted as components within broader tactical frameworks rather than independent indicators of team effectiveness.

## Conclusion

The high press defensive strategy has seemingly become prevalent within the most successful football teams in Europe. PPDA was devised as an indirect measure, a proxy of the high press, which has received face validity in the form of uptake by the main data providers and use by those in the football industry. The lack of scientific evaluation of PPDA required attention and this study presented the case for PPDA to be considered as a scale representing defensive tactics. Low PPDA values were associated with teams that enjoy a high proportion of possession playing against teams that tend to sit deep in a low block. This situation creates a relatively large number of ball losses and recoveries in the attacking area of the pitch, in which most of the players are located. This presents a good opportunity for a relatively large number of defensive actions per pass by the opponent; however, this is usually referred to as counter pressing and is distinct from the high press. Thus, the PPDA metric is likely to confuse, or combine, high pressing and counter pressing. The contrary situation exists for a team playing against a high possession opponent. They will typically attempt to play a direct style of football resulting in few players high up the pitch and therefore less opportunity to create a low PPDA value. On this basis, PPDA is reflective of playing style albeit the out of possession strategy which in turn is highly correlated to the in-possession style. PPDA was therefore only found to be a reasonable descriptive measure of a defensive playing style in situations where one team dominates possession. In more evenly contested matches PPDA values vary far less, both teams have similar values, with factors such as unsuccessful pressing, no attempts at high pressing or counter pressing being combined into the one PPDA metric to effectively obscure what was taking place. Future research should combine PPDA with spatial-temporal tracking metrics and artificial intelligence approaches to improve the understanding of the strengths and weaknesses of PPDA as an aid to tactical interpretation and made more practical if the different moments of the game (3) are separated for analyses.

## Data Availability

The data analyzed in this study is subject to the following licenses/restrictions: provided by Opta. Requests to access these datasets should be directed to https://www.statsperform.com/about/.
